# Near-total loss of buttressing stresses observed on Pine Island Ice Shelf, West Antarctica

**DOI:** 10.1073/pnas.2602994123

**Published:** 2026-08-11

**Authors:** Sarah Wells-Moran, Brent Minchew, Bryan Riel

**Affiliations:** ^a^ Department of Geophysical Sciences, Physical Sciences Division, University of Chicago, Chicago, IL 60615; ^b^ Department of Earth, Atmospheric, and Planetary Sciences, School of Science, Massachusetts Institute of Technology, Cambridge, MA 02139; ^c^ Seismological Laboratory, Division of Geological and Planetary Sciences, California Institute of Technology, Pasadena, CA 91125; ^d^Department of Geology, School of Earth Sciences, Zhejiang University, Hangzhou 310058, People’s Republic of China

**Keywords:** buttressing, ice shelf, collapse, Pine Island Glacier, West Antarctic Ice Sheet

## Abstract

Ice shelves, the floating extensions of the Antarctic Ice Sheet, provide critical buttressing stresses that resist the seaward flow of ice and help set the position of the grounding line, where the ice goes afloat. As buttressing stresses are diminished by thinning or fracturing and collapse of the ice shelf, glaciers tend to accelerate. Here, we focus on the response of Pine Island Ice Shelf (PIIS) in West Antarctica to multiple calving events and the disintegration of the lateral shear margins. Using observed time-series of the surface velocity fields between 2015 and 2024, we show multiple episodes of acceleration in ice flow and a marked reduction in the buttressing stresses. These observations show that PIIS experienced a significant reduction of its buttressing capacity during the observational record. We then investigate how a model glacier responds to loss in margin buttressing, and are able to broadly reproduce observations. By linking model simulations to observations, we recreate a timeline of buttressing loss on PIIS. These losses likely foreshadow a period of grounding line retreat and acceleration of Pine Island Glacier’s contribution to global mean sea level rise.

## Significance

Ice shelves provide a key source of stability to the Antarctic Ice Sheet by buttressing the flow of grounded ice. As ice shelves weaken and collapse due to rising temperatures, the glaciers buttressed by them speed up, increasing Antarctica’s contribution to sea level rise. Pine Island Glacier is the fastest flowing glacier in Antarctica and the ice shelf that buttresses it has undergone significant change in the last decade, resulting in a 20% increase in ice velocity. We compare observations to models to better understand the processes contributing to buttressing loss and conclude the Pine Island Ice Shelf has lost significant buttressing as the margins have deteriorated, destabilizing the grounding line and accelerating mass loss from West Antarctica.

Antarctic ice shelves—the floating extensions of the ice sheet—provide critical “safety bands” around the continent that moderate the flux of ice mass into the ocean through the buttressing effect they provide (1–3). When the Larsen B Ice Shelf collapsed in 2002, the glaciers buttressed by the shelf sped up two-to-six fold (4–6), emphasizing the important stabilizing role ice shelves play in ice sheet mass loss. Buttressing forces are especially important for the stability of the West Antarctic Ice Sheet (WAIS), which holds 5.3 m of sea level equivalent (7). Buttressing enables a marine ice sheet grounded on a retrograde bed, such as WAIS, to have a stable grounding line that would otherwise be vulnerable to rapid retreat in response to perturbations (2, 8). Despite the important role ice shelves play in modulating rates of sea level rise, the processes that control ice shelf stability and collapse are poorly understood due largely to a lack of observations, as only a handful of ice shelves have collapsed in the observational record (9–11). The lack of understanding about the future stability of the Antarctic ice shelf “safety bands” contributes significant uncertainty to sea level rise projections (12, 13).

We aim to disentangle the processes that contribute to ice shelf acceleration by investigating nearly a decade of observations over Pine Island Glacier as it has evolved in response to increasing ice damage. Pine Island Glacier (PIG) is the fastest flowing glacier in Antarctica (14) and is the largest contributor to sea level rise of all Antarctic glaciers (7, 15, 16). The Pine Island Ice Shelf (PIIS) buttresses 51 cm of sea level equivalent (7) and the glacier is grounded on a retrograde slope (17), leaving it vulnerable to irreversible retreat in response to perturbations. Between 1973 and 2008, ice shelf flow speed increased from 2.2 km/y to 4 km/y (18), accompanied by a 77% increase in ice discharge and increased ice speed detected throughout almost the entire PIG drainage area (18, 19). Flow speeds on the ice shelf remained relatively constant at 4 km/y from 2008 until 2017, when a large calving event occurred. After this calving event, flow speeds on PIIS steadily increased and reached a peak of 4.8 km/y in 2023, a 20% increase in speed over 6 y (20) and a 113% increase since 1973.

Increased ice velocity is often accompanied by grounding line retreat as the glacier thins to accommodate the increase in ice flux (3). Between 1992 and 2011, the PIG grounding line retreated more than 31 km (21–23). Since 2011, the PIG grounding line has only retreated a further 2 km at a steady rate (24). Ice shelf acceleration and grounding line retreat are likely closely linked to loss of buttressing through a variety of processes, including increased basal melt (25, 26), dynamic thinning, calving (20), and damage evolution (27–29).

## Past and Present Observations of Damage on PIIS

We primarily focus on damage development and evolution as a mechanism for buttressing loss, motivated by current observations of increasing damage in the PIIS Southern margin (29, 30) and historical records over PIIS of a cyclical process of increasing margin damage leading to calving front retreat dating back as far as 1972 (31). The processes that control damage formation and evolution are poorly constrained in natural glacier ice (32–35), but the effects of damage on the bulk material properties of ice are relatively well understood. As damage increases, the bulk viscosity of the ice decreases (36–38), causing areas that previously supplied a back stress on the ice shelf, such as shear margins or pinning points, to provide less resistance to flow, decreasing the buttressing force imposed by the ice shelf on the grounded ice upstream. We build on the previous literature by expanding the analysis of the observational record over PIG to 2025 and linking observations to idealized model responses to decreased margin viscosity as a proxy for increased damage and use these comparisons to create a timeline of buttressing loss on the PIIS over the last decade.

**Fig. 1.**
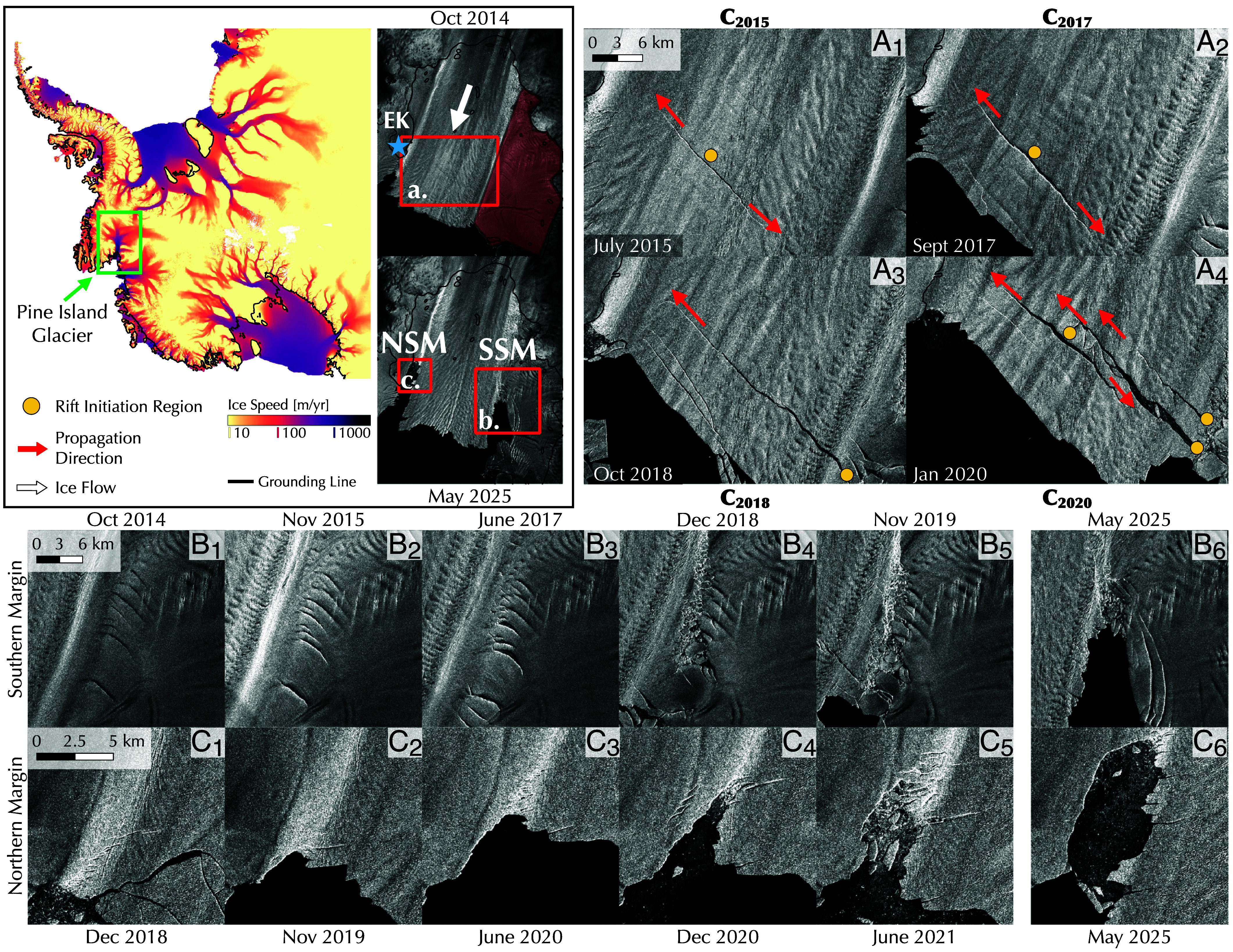
Evolution of damage on Pine Island Ice Shelf from 2014 to 2025. (*Top Left* Box) The location of Pine Island Glacier (PIG) on Antarctic ice velocity magnitude map (39). *Right*, Sentinel-1 images show the full extent of the Pine Island Ice Shelf (PIIS) in 2014 and 2025 with direction of flow denoted by a white arrow and the extents of (*A*–*C*) boxed in red. Location of Evans Knoll is marked with a blue star and the Southern Ice Shelf is shaded red. Grounding lines used in all observational figures are from 2022 (40, 41). (*A*) Modified Copernicus Sentinel-1 images of the PIIS just prior to each observed calving event. Rifts initiate in the general region of the yellow circles and propagate in the direction of the red arrows. A change in calving styles occurs between $C_{2017}$ and $C_{2018}$. Rifts $C_{2015}$ and $C_{2017}$ initiate in the center of the ice shelf and propagate laterally outward, whereas $C_{2018}$ initiates from the Southern Shear Margin (SSM) and propagates in one direction across the shelf, and $C_{2020}$ is formed by a system of rifts initiating in both the center and SSM. (*B* and *C*) Increasing damage and disintegration of the downstream portions of the SSM and Northern Shear Margin (NSM) as seen in Sentinel 1 imagery. ($B_{4-5}$ and $C_{4-5}$) show rapid fragmentation in the downstream SSM and NSM following $C_{2017}$ and $C_{2020}$, respectively. ($B_{6}$ and $C_{6}$) The state of the SSM and NSM as of May 2025. Between October 2014 and January 2026, the NSM and SSM lost approximately 40% and 45% of their initial lengths, respectively, while the current ice front is in almost the same position as 2014 (*SI Appendix*, Fig. S8).

We investigate changes in visible damage on PIIS between January 2015 and February 2024 using Sentinel-1A and 1B imagery. In this time period, we observe four calving events: $C_{2015}$ calves between July 25th and August 6th, 2015; $C_{2017}$ between September 21st and 23rd, 2017; $C_{2018}$ between October 25th and 29th, 2018; and $C_{2020}$ between February 7th and 9th, 2020 (Fig. 1*A*). The rifts that form $C_{2015}$ and $C_{2017}$ initiate in the center of the ice shelf, likely as basal crevasses (42), and propagate outward toward both shear margins as the rifts advect downstream. These rifts grow slowly and are visible for approximately two years before calving. We then observe a change in rifting styles, indicating a shift in stress states across the ice shelf. The $C_{2018}$ rift initiates in the Southern Shear Margin (SSM) and rapidly propagates across-flow toward the Northern Shear Margin (NSM) over the course of a month and a half. The calving style further changes for $C_{2020}$. Similarly to $C_{2015}$ and $C_{2017}$, the primary $C_{2020}$ rift initiates in the center of the ice shelf around 2017 and propagates outward as it advects downstream. As the initial $C_{2020}$ rift approaches the calving front, two rifts initiate in the SSM and propagate North toward the initial rift, creating a complex rift system that eventually forms $C_{2020}$. As of April 2026, no further large calving events have occurred.

In addition to the observed calving events, we see an increase in damage, defined as visible crevasses and melange, in both shear margins (Fig. 1 *B* and *C*). Between 2015 and 2017, existing rifts in the downstream SSM slowly widen. After $C_{2017}$, the existing rifts intersect and the downstream portion of the margin quickly deteriorates into melange, losing about 20% of its initial intact length over the course of just several months. Shortly after $C_{2020}$, the downstream portion of the NSM, which does not have an existing surface crevasse field as observed on the SSM, undergoes a similar rapid disintegration, losing 15% of its length between 2020 and 2026. From 2020 to 2023, melange continues to form from the collapsing NSM via increasing damage migrating upstream and breaking off small chunks of the ice shelf. In the NSM, this melange evacuates the area at a steady rate and fully evacuates to open water by December 2023 when the intact ice stops producing further melange. In contrast, the melange produced by the collapse of the downstream SSM is trapped between the main trunk and the PIG Southern Ice Shelf for several years after $C_{2017}$, resulting in a brief period of recoupling between the PIG Southern Ice Shelf and main trunk of PIIS when the melange is trapped within the margin by landfast sea ice (43). The SSM melange begins evacuating in March of 2024 when a piece of ice on the PIG Southern Ice Shelf breaks away and unplugs the path between the melange and open ocean (*SI Appendix*, Fig. S1). As the main shelf loses contact with the PIG Southern Ice Shelf and melange, the calving front shifts southward.

## Response of PIIS to Increasing Damage

**Fig. 2.**
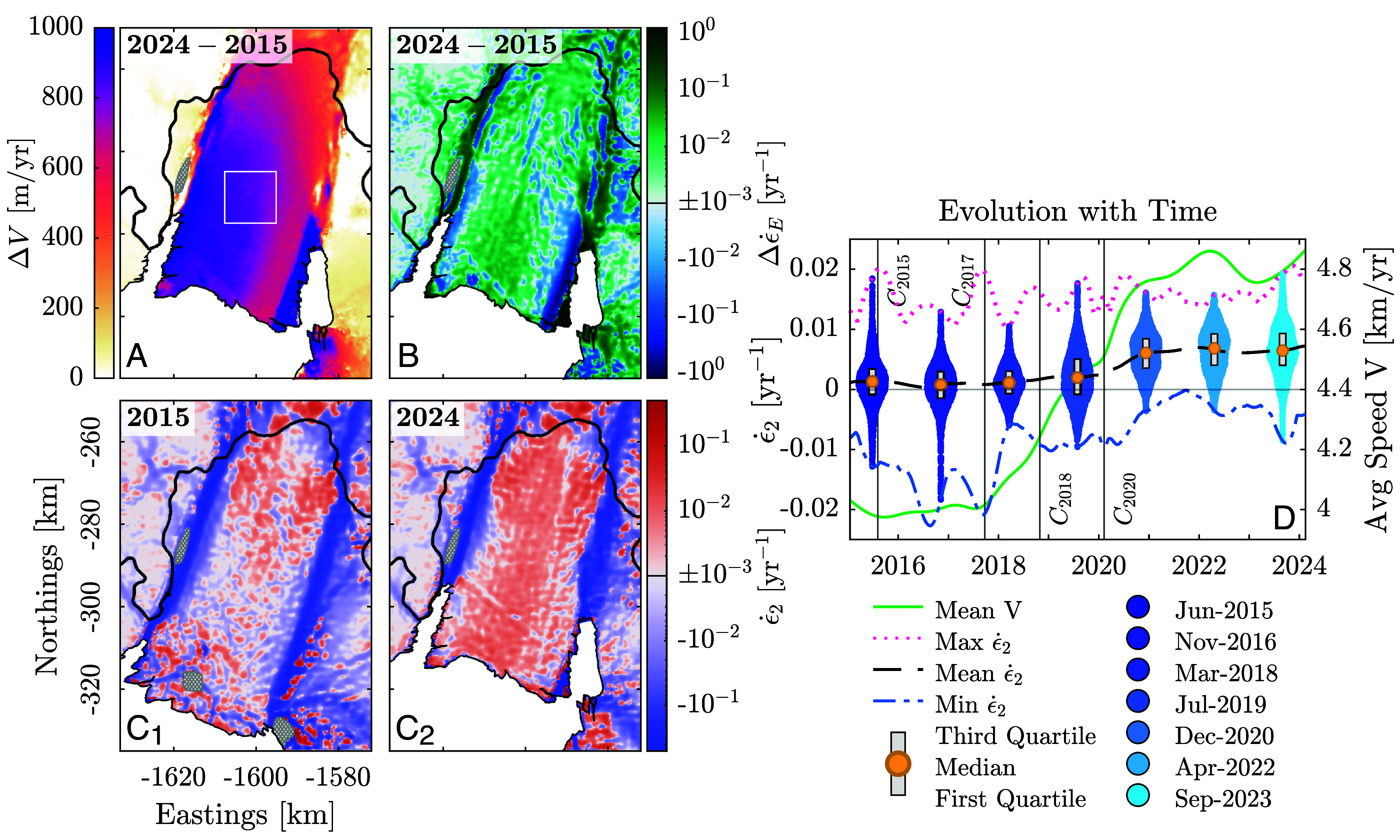
The change of observed (*A*) ice speed (ΔV) and (*B*) effective strain rate ($\Delta {\dot{\epsilon }}_{E}$) fields on PIIS between January 2015 and February 2024. While average ice speed increases over the observational record, it does not increase uniformly across flow. (*C*) Least principal strain rate (${\dot{\epsilon }}_{2}$) fields in (1) 2015 and (2) 2024, which show a marked increase in the area of PIIS in pure tension. Regions with no data are covered with hatched shading. We manually trace calving fronts from satellite imagery (44). (*D*) Evolution over time of average ice speed and least principal strain rate values sampled from the 156.25 km^2^ region outlined by a white box in (*A*). Black vertical lines denote calving events. Average ice shelf speed (solid green line) increases closely correlated to $C_{2017}$ and continues increasing until 2023. We plot the maximum, mean, and median values of ${\dot{\epsilon }}_{2}$ in the outlined area. Additionally, we provide violin plots of ${\dot{\epsilon }}_{2}$ at 7 dates. Prior to 2020, the first quartile of the sampled ${\dot{\epsilon }}_{2}$ values are negative. After $C_{2020}$, more than 75% of the sampled ${\dot{\epsilon }}_{2}$ values are greater than 0.

We aim to link visible changes in ice shelf damage to observable changes in velocity and strain rates. We develop 1,000 time-dependent velocity fields with a time step of ∼3 d (*Velocity Fields*) over PIG from January 2015 to February 2024 and from the velocities derive effective and principal strain rate fields, following the convention of ${\dot{\epsilon }}_{1}>{\dot{\epsilon }}_{2}$ and positive values denoting tension. Thus, the ice shelf is in a purely tensile regime when ${\dot{\epsilon }}_{2}>0$ as ${\dot{\epsilon }}_{1}$ must necessarily be positive by convention. For this reason, we analyze changes in tensile regimes on the ice shelf via the least principal strain rate ${\dot{\epsilon }}_{2}$. For each field, we calculate mean, minimum, and maximum velocities and strain rates within a 156.25 km^2^ area (51 × 51 pixels) in the center of the ice shelf, outlined in Fig. 2*A*, which we use to represent the average state of the ice shelf.

**Fig. 3.**
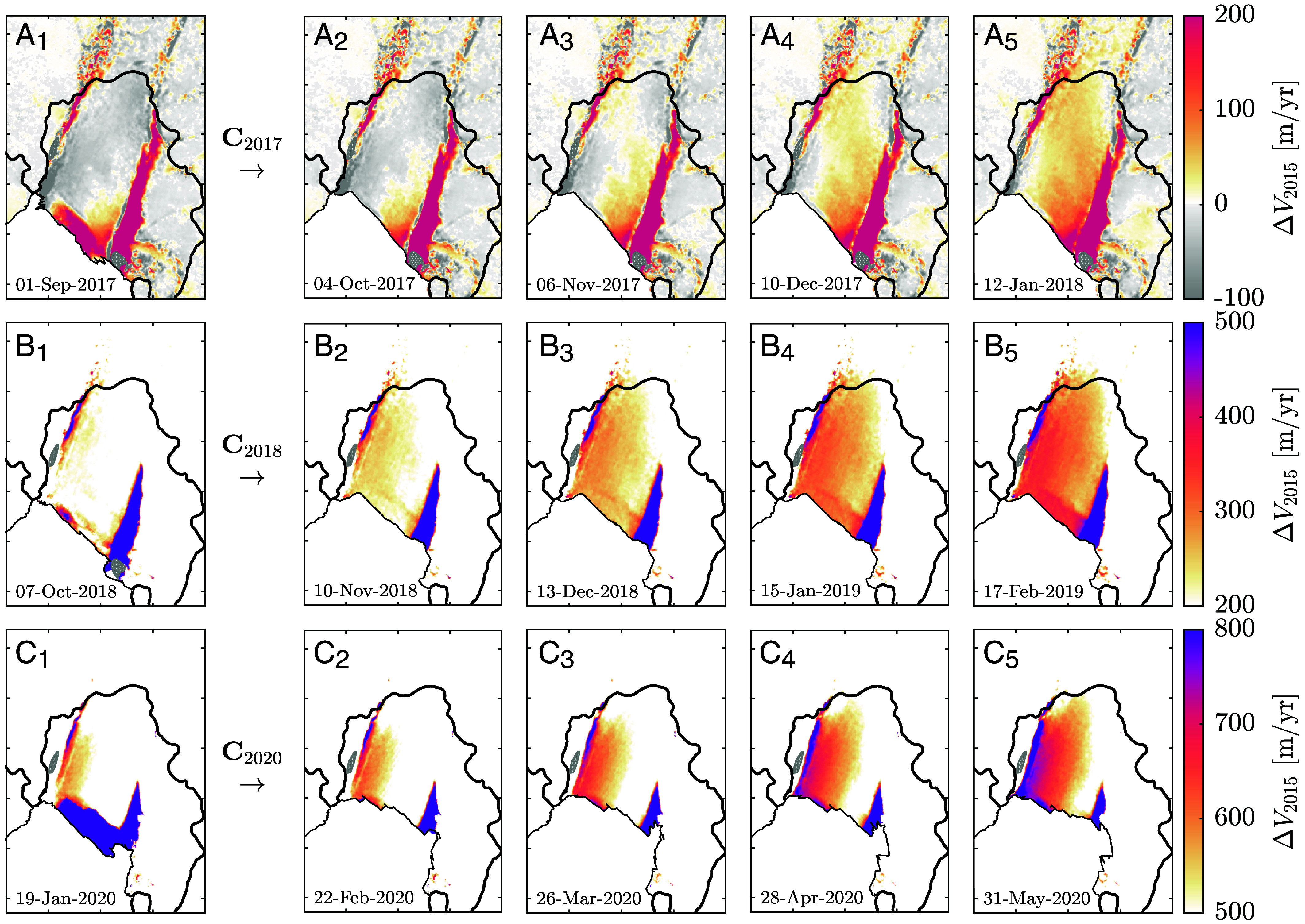
Change in ice speed relative to January 2015 (1) 3 wk preceding and (2 to 5) in the 16 wk following (*A*) $C_{2017}$, (*B*) $C_{2018}$, and (*C*) $C_{2020}$. Each panel is separated by approximately 33 d. Colorbars start at different ΔV relative to 2015 but all span 300 m/y to allow for consistent comparison of smaller across-flow speed changes. After $C_{2017}$, increased velocities propagate from the downstream edge of the SSM toward the upstream portion of the NSM. After $C_{2018}$ and $C_{2020}$, increased velocities propagate across flow from the NSM toward the SSM. Average velocities on the ice shelf increase by approximately (*A*) 65 m/y, (*B*) 125 m/y, and (*C*) 152 m/y in the 132 d between (1) and (5). We manually trace the ice front location from Sentinel-1 imagery.

The mean speed of the ice shelf increases by almost 900 m/y between 2015 and 2024, the majority of which occurs between 2017 and 2022 (Fig. 2 *A* and *D* and *SI Appendix*, Fig. S2 and Movie S1). Ice upstream of the grounding line also speeds up in this time frame (45), but we limit our analysis to changes occurring in the shelf region. The ice shelf begins speeding up in April 2017, 5 mo prior to $C_{2017}$, and begins slowing down after March 2022. We observe another increase in speed in February 2023 and speeds continue increasing through the end of our observational data. The maximum rate of acceleration occurs around April 2020, two months after $C_{2020}$, and the next two largest accelerations occur one month after $C_{2018}$ and three months after $C_{2017}$, respectively. The 900 m/y speed-up does not occur uniformly across flow (Fig. 3). The 2017 speed-up initiates in the SSM close to the calving front, and increased speeds propagate diagonally toward the region of the NSM close to the grounding line. After $C_{2018}$, the speed-up originates from the NSM and propagates laterally across the ice shelf. The third major speed-up initiates from the downstream NSM in 2020, partially as a continuation of the increase associated with $C_{2018}$, which $C_{2020}$ accentuates and accelerates. The magnitude of speed-up in the 132 d following calving increases for each successive calving event with the exception of $C_{2015}$.

In contrast to the increase in ice flow speed, which has one main period of speed-up between 2017 and 2022, we observe two distinct periods of increasing strain rates (*SI Appendix*, Fig. S2 and Movie S2). Between $C_{2017}$ and $C_{2020}$, principal and effective strain rates gradually increase. After $C_{2020}$, strain rates sharply increase, and then level out around January 2021 before starting to decrease in December 2021. The maximum rate of increase in both velocity magnitude and strain rates occurs in close proximity to $C_{2020}$ (*SI Appendix*, Fig. S2). After $C_{2020}$, the ice shelf shifts to an almost entirely tensile regime, defined by ${\dot{\epsilon }}_{1},{\dot{\epsilon }}_{2}>0$ (Fig. 2 *C* and *D* and *SI Appendix*, Fig. S3 and Movie S4). Prior to $C_{2020}$, between 25% and 50% of ${\dot{\epsilon }}_{2}$ values sampled are negative and the mean of ${\dot{\epsilon }}_{2}$ is $1.3\times 10^{-3}$ ${\text{y}}^{-1}$, whereas after $C_{2020}$, less than 25% of sampled ${\dot{\epsilon }}_{2}$ are negative and the mean of ${\dot{\epsilon }}_{2}$ more than quadruples to $5.8\times 10^{-3}$ ${\text{y}}^{-1}$ (Fig. 2*D*).

Strain rate fields show the SSM migrates 5 to 10 km outward from the trunk of the ice shelf between 2015 and 2018 (*SI Appendix*, Fig. S4), concomitant to our observations of increasing damage within the SSM and additionally noted in other studies (30). As we are primarily interested in how PIIS is changing in response to these observations of damage, we subtract the January 2015 ${\dot{\epsilon }}_{E}$ field from subsequent ${\dot{\epsilon }}_{E}$ fields and refer to the resulting fields as $\Delta {\dot{\epsilon }}_{E}$. In $\Delta {\dot{\epsilon }}_{E}$, we note a distinctive pattern that we hypothesize is associated with margin weakening (Fig. 2*B* and *SI Appendix*, Fig. S4 and Movie S3). This is especially visible when observing the evolution of the SSM. As the margin migrates, strain rates in the old margin decrease relative to 2015, while strain rates along the path of the new margin increase. This leaves a pair of adjacent along-flow stripes of increasing and decreasing strain rates along the boundary between the old and new margins. We find a similar pattern of weakening in the upstream NSM strain rate fields which starts near a surface crevasse field near the grounding line (*SI Appendix*, Fig. S5) and grows downstream as damage is advected toward the calving front. Mechanistically, this pattern can be explained by changes in viscosity: As damage within the margin increases, bulk viscosity decreases, resulting in a steeper velocity gradient concentrated through damaged areas. As strain rates increase, this can also generate new damage which further reduces the bulk viscosity of the shear margin ice (29, 35). This feedback loop of damage generation may explain why we find the strain rate pattern associated with margin weakening in the NSM without observed margin migration. As viscosity within a damaged region decreases, the shear margin will follow the path of least resistance and narrow as the deformation is concentrated in an increasingly damaged band of ice.

Thwaites Glacier, a neighbor of PIG in the Amundsen Sea Embayment (ASE), provides an analog for some of the processes described above. Benn et al. (46) observe a similar phenomenon of damage concentration and strain localization within a shear zone on the Thwaites Eastern Ice Shelf in 2020 and describe the damaged shear zone as the “weakest link” allowing the eastern portion of ice shelf to accelerate as the weak shear zone decoupled the shelf from a pinning point. Banerjee et al. (47) build on that work by identifying a velocity perturbation propagating outward from the shear zone correlated with a period of increasing ice shelf flow speed. The similarities between these observations and the changes we observe on PIIS further points toward margin weakening and collapse as an explanation for increasing ice shelf flow speed.

## Model Simulations

**Fig. 4.**
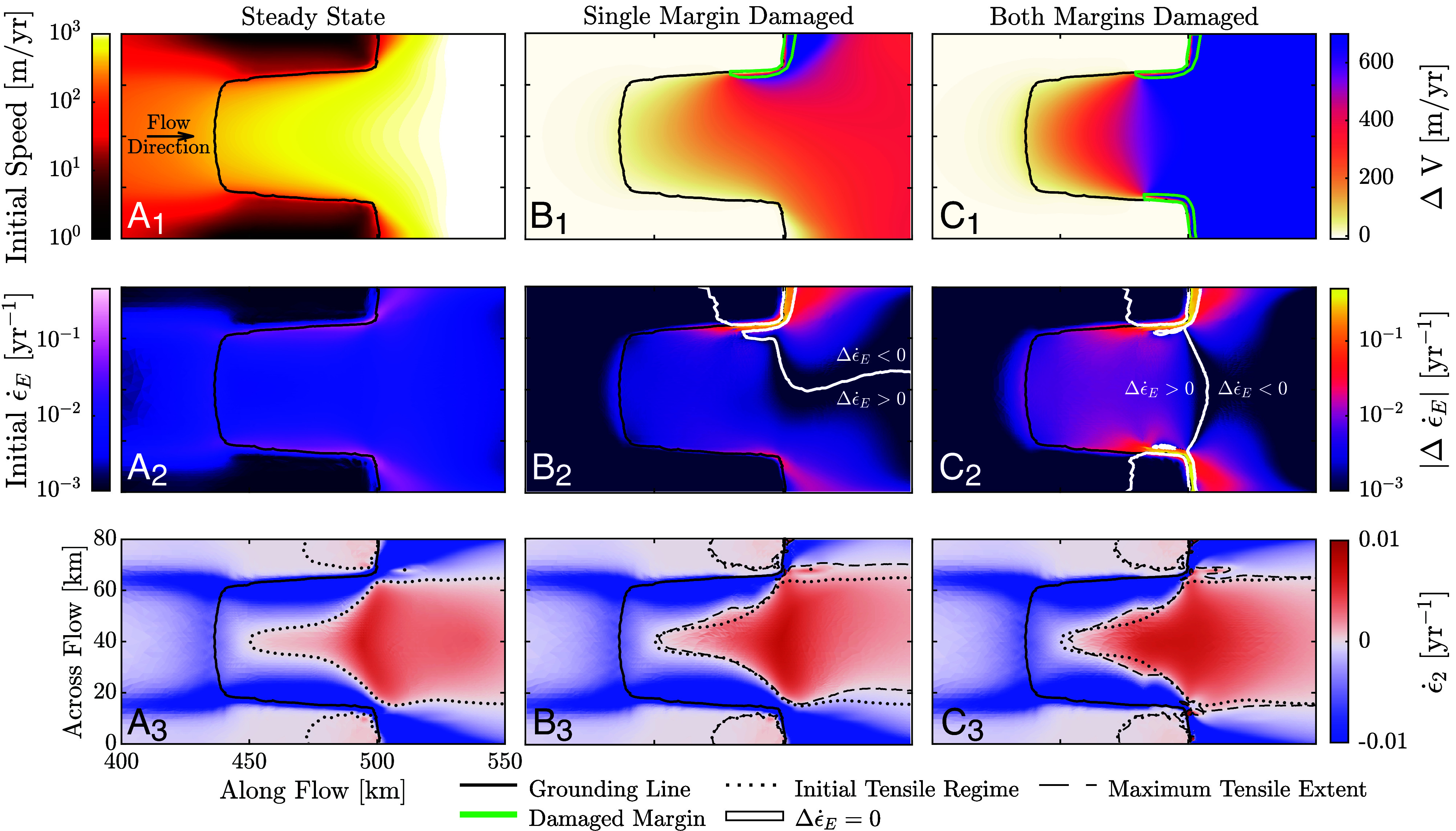
Ice sheet model results for (*A*) the steady state glacier after model spin up, (*B*) the instantaneous response after weakening a single margin, and (*C*) the instantaneous response after weakening both margins. (*B*_1_ and *C*_1_) show changes in modeled speed relative to (*A*_1_), with the area of weakened margin outlined in green. (*B*_2_) and (*C*_2_) show changes in the magnitude of modeled effective strain rate relative to (*A*_2_), with a solid white line separating regions of increasing and decreasing strain rates relative to (*A*_2_) steady state. (*B*_3_) and (*C*_3_) show the modeled ${\dot{\epsilon }}_{2}$ field, with red denoting areas in pure tension (${\dot{\epsilon }}_{1},{\dot{\epsilon }}_{2},>0$). The steady state tensile extent is plotted as a dotted line on both (*B*_3_) and (*C*_3_) to better visualize change in tensile extent between each scenario.

To better contextualize these observations of change on PIIS, we investigate the response of a simple model ice shelf to induced margin damage. We utilize the Ice-Sheet and Sea-level System Model (ISSM) (48) and spin up an idealized glacier to steady state following the MISMIP+ geometry, methodology, and parameters (49), which is qualitatively based in the PIG geometry. Once the model reaches steady state, we simulate damage in the ice shelf margin by decreasing the ice viscosity coefficient (rigidity parameter) by an order of magnitude and then calculate the instantaneous stress response of the model to the imposed damage. We investigate two scenarios of buttressing loss via induced damage: 1) Damaging the downstream edge of one model margin, and 2) damaging both margins. The region of imposed damage for both scenarios is outlined in green on Fig. 4*B*_1_ and *C*_1_.

Similarly to our analysis of the observations, we subtract steady state (Fig. 4*A*) from modeled instantaneous changes in ice speed and ${\dot{\epsilon }}_{E}$. In both model simulations, we observe a large increase in ${\dot{\epsilon }}_{E}$ within the damaged margin and a decrease in ${\dot{\epsilon }}_{E}$ directly adjacent to the damaged margin. When only one margin is damaged, strain rates also increase on the downstream corner of the opposite, intact margin (Fig. 4*B*_2_). Additionally, in the single margin scenario, instantaneous flow speed increases across flow and the region of pure tension expands toward the damaged margin. When both margins are damaged, speed increases symmetrically across flow and the largest increase in speed is along the center line, and the region of the model in pure tension widens to encompass a larger area in the center of the ice shelf.

## Reconstructing a Timeline of Buttressing Loss

**Fig. 5.**
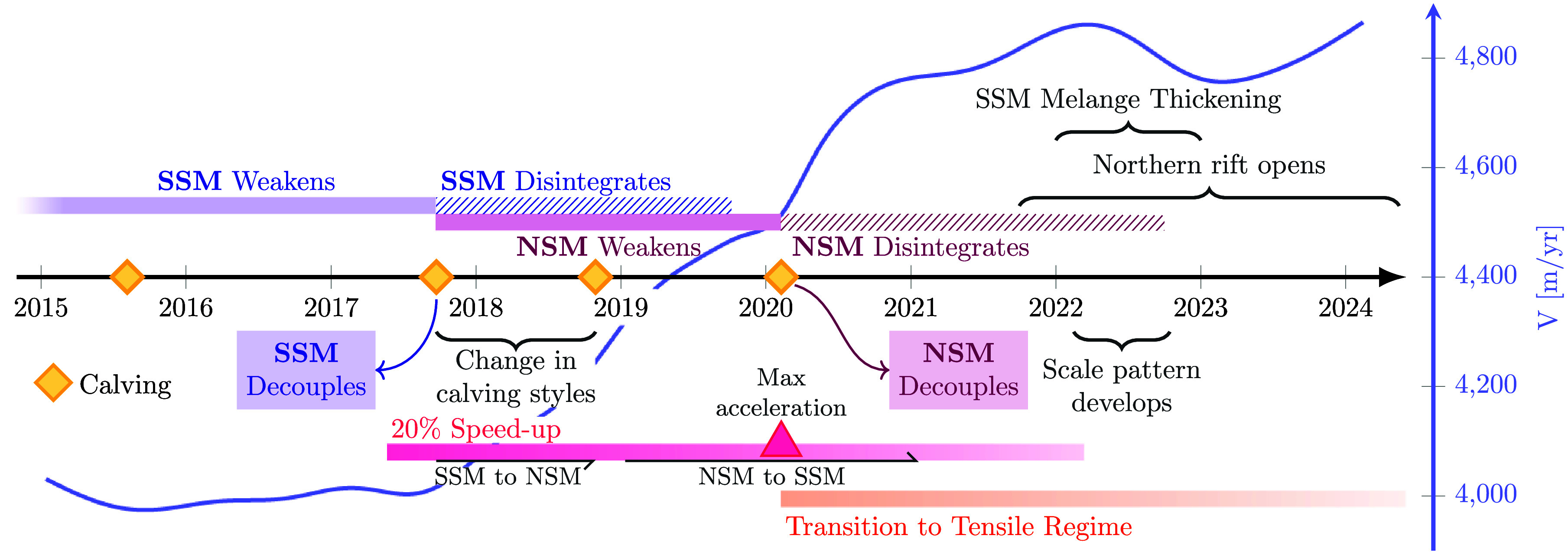
Proposed timeline of buttressing loss on PIIS. Average velocity magnitude is plotted in the background in light blue.

We compare our model simulations to observed changes on PIIS to construct a timeline of buttressing loss (Fig. 5). Between 2015 and 2024, the majority of buttressing is lost via the ice shelf decoupling from its shear margins via damage development and calving. Damage is present in the SSM prior to 2015 and increases over time (Fig. 1*B*), thus limiting the ability of the margin to resist ice flow. As seen in both simulations (Fig. 4 *B* and *C*) and observations (Fig. 2*B* and *SI Appendix*, Fig. S4), effective strain rates increase within damaged regions, which promotes further damage development and weakening (29, 35). $C_{2017}$ then reduces the area of ice shelf in contact with the margins, reducing lateral drag and enabling increased ice flow speed across the ice shelf. Following $C_{2017}$, the downstream SSM rapidly fractures into melange (Fig. 1*B*_4,5,6_). Our model simulations show a significant speed-up originating from the decoupled region, which we observe occurring directly correlated with $C_{2017}$ (Fig. 3*A*). $C_{2017}$ marks the start of the 20% increase in observed velocities over a period of 5 y.

As the downstream SSM decouples from the trunk of the ice shelf, velocities near the SSM increase and induce rotation about the downstream corner of the NSM. This increases tensile stresses near the SSM which promotes rift initiation in the margin and propagation northward, forming $C_{2018}$. In our simulations, loss of buttressing along a single margin increases strain rates about the opposite margin (Fig. 4*B*). Increased strain rates about the NSM promote damage development within the margin, resulting in further weakening (*SI Appendix*, Fig. S4). As the NSM begins to weaken, ice speed continues to increase. After $C_{2018}$, the speed-up is concentrated along the entire NSM (Fig. 3*B*).

$C_{2020}$ removes a portion of ice shelf that was grounded near Evans Knoll. The ice accelerates at approximately 1 m/y/d after $C_{2020}$ and peaks at a rate of 1.4 m/y/d 60 d after calving (*SI Appendix*, Fig. S2). Following $C_{2020}$, the downstream NSM fractures and turns into melange over the course of a year (Fig. 1*C*), similarly to the breakup of the downstream SSM following $C_{2017}$. After 2020, the majority of the ice shelf shifts to a tensile regime defined as ${\dot{\epsilon }}_{2}>0$ (Fig. 2 *C* and *D* and *SI Appendix*, Fig. S3), which we replicate in model simulations when removing buttressing from both margins (Fig. 4*C*_3_). The speed-up following $C_{2020}$ originates from the NSM (Fig. 3*C*). After $C_{2020}$, no further calving events have occurred as of April 2026.

**Fig. 6.**
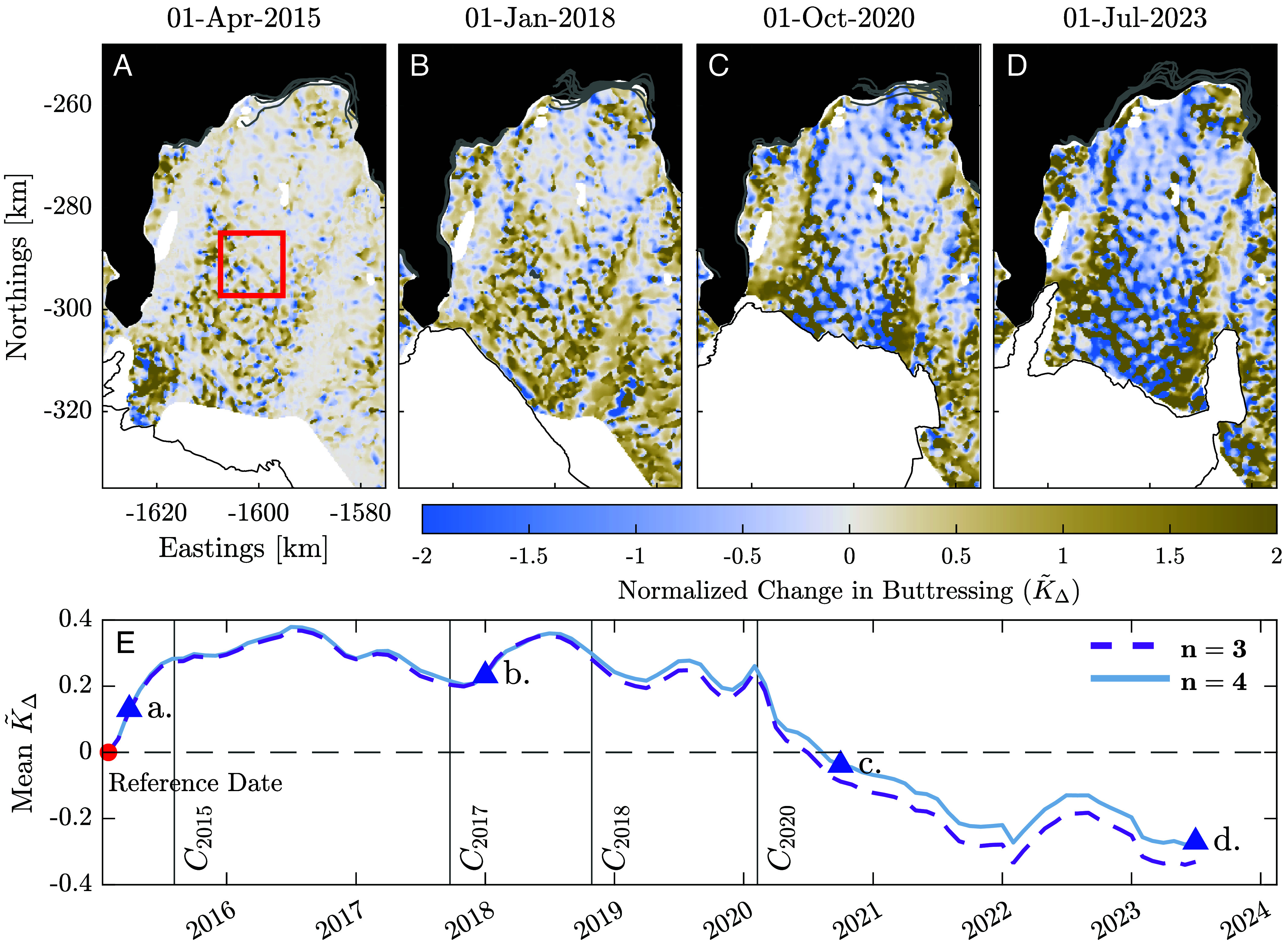
(*A*–*D*) Normalized change in buttressing (${\tilde{K}}_{\Delta }$) on PIIS from 2015 to 2023, calculated relative to 2015 as described in *Quantifying Buttressing Loss*. Grounded ice is masked out in black using 2022 grounding lines, and we plot DINSAR-derived grounding lines (24) for each year as gray lines on top of the grounded ice mask. (*E*) Mean ${\tilde{K}}_{\Delta }$ relative to 2015 over time. Time points of (*A*–*D*) are plotted as blue triangles. There is relatively little difference between ${\tilde{K}}_{\Delta }$ calculated using n=3 vs. n=4. Additional plots of ${\tilde{K}}_{\Delta }$ relative to 2017 and 2023 are available in *SI Appendix*, Figs. S6 and S7.

To further our comparison between simulations and observations, we calculate normalized change in buttressing (${\tilde{K}}_{\Delta }$) over PIIS using thickness data from 2012 to 2023 derived by ref. 50. In defining ${\tilde{K}}_{\Delta }$, we leverage the temporal resolution of our data to eliminate most rheological uncertainties from calculations of buttressing, which we describe in more detail in *Quantifying Buttressing Loss*. We find a substantial drop in ${\tilde{K}}_{\Delta }$ for every tested reference date directly after $C_{2020}$ (Fig. 6 and *SI Appendix*, Figs. S6 and S7), bolstering our theory that the collapse of the downstream NSM resulted in substantial loss of buttressing on PIIS. Additionally, we find a small decrease in ${\tilde{K}}_{\Delta }$ preceding $C_{2017}$, correlated with increasing damage in the SSM. Despite the quantity of damage in the SSM, buttressing increases between $C_{2017}$ and $C_{2018}$, corroborating the findings of Savidge et al. (30) that coupling between PIIS and the PIG Southern Ice Shelf existed until $C_{2018}$ removed a portion of ice connecting the two. Between 2022 and 2023, there is a small increase in buttressing corresponding to the suggested dates of melange coupling between PIIS and the PIG Southern Ice Shelf proposed by Chien et al. (43) and the dip in flow speed observed in that time frame.

## Open Questions

This timeline leaves several outstanding questions:

### What Portion of Each Margin Has Lost the Ability To Support the Shear Stresses That Provide Buttressing?

The SSM appears visually damaged along its entire length, but portions of the NSM do not appear as damaged, although there are regions near the grounding line within both margins that generate visible damage that is advected down the margin with ice flow (*SI Appendix*, Fig. S5). While we can say for certain that the downstream regions of the former margins that are now open ocean have collapsed, it is difficult to quantify the effect advected damage has had on the remaining margins. Both margins developed patterns in $\Delta {\dot{\epsilon }}_{E}$ fields that signify weakening along the entire margin and strain localization, and the three year time offset between decoupling in the SSM and NSM may have intensified stresses on the opposite margin, thus generating more damage. The entire remaining lengths of both margins are likely weaker than they were in 2015, but we cannot definitively quantify how much buttressing potential they have lost as a result. As of April 2026, PIIS extends almost twice the length of the remaining margins into open ocean, floating as an unbuttressed ice tongue for half its length (*SI Appendix*, Figs. S8 and S9). If no further calving events occur, the calving front may make contact with the Western edge of the PIG Southern Ice Shelf within the next year and a half, which could either stabilize the ice or generate even more damage within the already damaged ice shelf.

### How Did This Process of Buttressing Loss Initiate?

Due to the continuous history of change over PIIS and the limited observational record prior to the launch of Sentinel-1, it is difficult to fully disentangle when this process initiated, although these limited observations can give us insight into some of the processes involved. Over the satellite observational record, PIG has experienced three phases of acceleration from 1974 to 1987, 1994 to 2010, and 2017 to 2022 (18, 20, 30, 51–53). The first two phases of acceleration were driven by ice shelf thinning as a result of Circumpolar Deep Water (CDW) intrusion (52, 54–57) coupled with the PIG grounding line positioned on a bathymetric high just in front of a large, flat ice plain (58). Warmer waters eroded the ice at the grounding line and unpinned it from the bathymetric high, resulting in almost 31 km of grounding line retreat along the centerline and 7 to 10 km along the sides between 2005 and 2009 (21). As a result of decreased resistive stresses and increasing flow speed, damage within the PIIS margins increased. Thinning may precondition damage production within the margins as thinner ice is more likely to fracture rather than viscously flow. Bindschadler (31) investigates the history of margin damage from 1973 to 2002 and notes the progression of ice from flowing over pinning points to fracturing over them, which then decouples the shelf from its margins as the damage advects downstream. In PIG and other ASE glaciers, margin retreat has historically preceded calving front retreat (59).

### Did Ocean-Induced Ice Shelf Thinning Also Cause the Most Recent Speed-Up?

Ice shelf mass change is strongly linked to climactic variability, especially El Niño/Southern Oscillation (ENSO) (55, 60–63). 2015–2016 was classified as a very strong El Niño event by the Relative Oceanic Niño Index (RONI) (64), although there are no moderate or larger El Niño events from 2017 to 2022. While the very strong El Niño event could have been a catalyst for the timeline of buttressing loss proposed above, several modeling studies show that ice shelf thinning alone is not enough to reproduce observations of ice shelf acceleration from 2017-present (20, 28, 53, 65). Even the influence of anthropogenic forcing is only able to account for approximately 20% of PIG grounding line retreat from the 1940s-present (66). Given the close correlation between calving events and the observed increases in flow speed (Fig. 2*D* and *SI Appendix*, Fig. S2), we suggest the primary driver of the 2017–2022 acceleration is calving and damage. Rates of thinning have slowed across most ASE glaciers starting around 2008 (67), further indicating that change in ocean temperatures is not likely driving this recent period of acceleration. This does not negate the important influence of basal melt on ice shelf stability. Prior acceleration, basal melting, and ice shelf thinning likely primed the ice shelf for further damage production within the margins.

### To What Extent Did Pinning Points Contribute to the Observed Dynamic Changes of PIIS?

Between 2015 and 2024, there are no major permanent pinning points in contact with the center of PIIS that would influence the dynamic response of the ice shelf during the most recent period of acceleration (50, 68, 69). $C_{2015}$ may have initiated due to intermittent contact with an ice ridge, but the ice shelf has had no further contact with the ridge after $C_{2015}$ (69). There is evidence of ephemeral grounding, where the pinning point connects with the base of the ice shelf only at low tide, at a pinning point in the center of the ice shelf (20, 70). However, ephemeral grounding events are modeled to have negligible impacts on ice shelf velocity fields (71) and are instead a better metric of changes in ice thickness. After $C_{2020}$, ephemeral grounding events recorded by Chien et al. cease for almost a year, indicating thinning of the ice shelf in response to the observed loss in buttressing. These ephemeral grounding events may have contributed to the production of the three large basal crevasses which eventually form into the full thickness rifts which form $C_{2015}$, $C_{2017}$, and $C_{2015}$, thus it is important to understand the role ephemeral grounding plays in large-scale rift development (20, 70).

Pinning points located within or near the shear margins have a much more immediate impact on loss of buttressing. Several rifts that result in large calving events initiate downstream of damage in the shear margins (72), including $C_{2018}$ and one of the rifts that formed $C_{2020}$. This shear margin damage develops as the ice shelf initially accelerates in response to ocean thinning. Damage development sets in motion a positive feedback loop where damage weakens the shear margins, resulting in further acceleration, damage production, and weakening (29, 35). This cycle has been observed within the margins of PIIS from 1973-present (27, 31, 59). Additionally, we find the lopsided nature of margin destabilization may exacerbate damage development on the opposite margin. As one margin decouples, stresses about the opposite margin increase, promoting damage development. Our observations are potentially part of a longer process of margin destabilization switching between the shear margins that has been ongoing since before the 1970s, almost like unzipping the margins of the ice shelf. The key question is what causes a pinning point to transition from providing buttressing to producing damage? We theorize thinning is an important mechanism, but further study is necessary to tease out the interplay of thinning and damage surrounding margin pinning points.

## Implications for the Stability of Pine Island Glacier

We present a timeline of observed buttressing loss on Pine Island Ice Shelf over the course of a decade. Ice shelf buttressing sets the stability of PIG and its loss portends a period of irreversible ice loss and subsequent sea level rise (2, 3, 73). The rate at which PIG will now contribute to sea level rise is a source of deep uncertainty (13) and depends on a variety of factors, including ocean-driven melting (74), the mechanics and hydrology of the glacier bed (75), and the viscosity of ice (76, 77). The total loss and contribution to sea-level rise has been estimated in previous work that evaluates tipping points and early warning indicators (78). These authors identify three grounding line positions as tipping points, beyond which ice loss becomes self-sustaining until the grounding line reaches another point of stability. The current grounding line position approximates one of the tipping point positions, and we hypothesize that the grounding line will retreat in response to the loss of buttressing and 20% increase in ice shelf flow speed. During the last phase of rapid grounding line retreat, PIIS experienced a 1.8 km/y increase in ice shelf flow speed (21). Between 2011 and 2025 the grounding line retreated 2 km (24). As grounded ice speeds up in response to the almost 900 m/y increase of ice shelf speed between 2017 and 2022, thinning must occur to accommodate the increased flux of ice across the grounding line, and thus grounding line retreat is highly likely. As of this writing, there is no definitive evidence that such a retreat in response to the 2017 speed-up has begun, likely due to the mechanical properties and topography of the bed near the grounding zone (79). Continued observations of grounding line migration will yield further insights into the processes that set the persistence of grounding line positions in PIG.

While the future of PIG is unclear, there are some notable observations that may elucidate how PIIS will evolve. The current ice front has developed a scale-like texture which is very similar to the texture of the Thwaites Eastern Ice Tongue prior to its eventual disintegration (*SI Appendix*, Fig S9 *B* and *C*), and also resembles patterns present on other ice tongues across Antarctica. This pattern has likely developed due to melt water channelization under the ice shelf and the viscous relaxation of ice surrounding the channels, but the length scale of these features may give us insights into a material property of ice. Additionally, we note a rift growing near the grounding line of the NSM (*SI Appendix*, Fig. S9*A*). If this rift continues to grow across the ice shelf, we may see a significant loss of ice shelf area within the next decade.

## Materials and Methods

### Velocity Fields.

We interpolate velocity products processed by the Greenland Ice Mapping Project (80) by fitting the velocity time series on a pixel-by-pixel basis with a collection of integrated B-splines (81). This approach allows for flexible reconstruction of temporal variations with timescales ranging from half a year to two years. Once fitted, we evaluate the B-splines on a uniformly spaced time grid to create 1,000 time-dependent velocity fields over PIG from January 2015 to February 2024.

From the velocity fields, we derive strain rates as the symmetrical component of the horizontal velocity gradient tensor, which we compute at each pixel using a 4th-order Savitzky–Golay filter with a window size of 2.5 km. This window size was chosen to prioritize preservation of strain rate magnitudes and spatial patterns while minimizing the influence of velocity noise. From the strain rate tensor at each location, we calculate the principal horizontal strain rates from the tensor eigenvalues, as well as the effective strain rate computed as the square root of the second tensor invariant ${\dot{\epsilon }}_{E}=\sqrt{({\dot{\epsilon }}_{1}^{2}+{\dot{\epsilon }}_{2}^{2})/2}$.

### Model Setup.

We implement the third phase of the Marine Ice Sheet Model Intercomparison Project (MISMIP+) (49) in the Ice-sheet and Sea-level System Model (ISSM) (48). MISMIP+ is qualitatively based on the geometry of PIG and explicitly accounts for lateral buttressing, which is not incorporated in the first two phases of the MISMIP experiments (82). The MISMIP+ model domain is 680 km along flow and 80 km across flow, and uses the Shallow Shelf Approximation (83) to simplify and solve the Stokes Equations, which averages values with depth, resulting in a 2-dimensional model. We run the model forward on a uniform mesh for 20,000 y to achieve steady state, then refine the mesh around the grounding line and ice shelf using ISSM grid refinement functions. In steady state, our model grounding line falls approximately 440 km from the start of the model domain.

In continuum models, damage of all scales is represented as a state variable, *D*, that ranges from 0 (undamaged) to 1 (most damaged) and modifies the bulk viscosity of the material (37, 38, 84). Material properties are linked to flow properties within the Full Stokes Equations through constitutive relations. Ice is a non-Newtonian fluid that is well approximated using Glen’s Flow Law (85):

$$\begin{array}{c} \tau_{\mathrm{ij}}=2\eta {\dot{\epsilon }}_{\mathrm{ij}}, \end{array}$$

where $\tau_{\mathrm{ij}}$ is the deviatoric stress tensor, ${\dot{\epsilon }}_{\mathrm{ij}}$ is the strain rate tensor, and *η* is the dynamic viscosity, defined as

$$\begin{array}{c} \eta_{\mathrm{damage}}=\left(1-D\right)\frac{B}{2{\dot{\epsilon }}_{e}^{(n-1)/n}}, \end{array}$$

where *D* is the damage state variable, *B* is the ice rigidity, ${\dot{\epsilon }}_{e}$ is the effective strain rate, and *n* is the flow law exponent. In this work, we assume n=3 for consistency with other MISMIP+ experiments.

Due to model constraints and to simplify computation, we choose to modify the value of ice rigidity, *B*, to simulate decreases in viscosity related to damage rather than implementing an evolving state damage variable D. Ice rigidity is commonly used in ice flow modeling instead of the ice softness parameter *A* typically used in the Glen-Nye flow law and is equivalent to $B=A^{-1/n}$. We utilize an empirically derived value of $B_{\mathrm{intact}}={\left(6.338\times 10^{-25}\right)}^{-1/3}=1.164\times 10^{8}$ Pa s^1/3^ for the general ice shelf, consistent with the initial value of *A* in the MISMIP+ methodology (49), and an arbitrarily chosen value of $B_{\mathrm{damage}}=2.0\times 10^{7}$ Pa s^1/3^ for the damaged shear margins, corresponding to a value of D≈0.9.

From the steady state model, we set $B=B_{\mathrm{damage}}$ within the shear margins (highlighted in green on Fig. 4) and calculate the instantaneous stress response of the model to this change in buttressing. We do not run the model forward after perturbation due to the relatively quick timescale over which the observed changes occur and due to uncertainties involved with modeling transient properties such as ice thickness, damage evolution, and grounding line migration (86). We also do not prescribe a basal melt rate since we do not run the model forward after reaching steady state. We focus entirely on changes within the buttressed floating ice shelf, as the Shallow Shelf Approximation makes assumptions that do not well-approximate grounded ice.

### Quantifying Buttressing Loss.

Previous studies have quantified buttressing as a nondimensional number representing the difference between the vertically integrated pressure exerted by the ocean on the ice column and the vertically integrated stress within the ice column (1, 2, 87, 88):

$$\begin{array}{c} K_{n}=1-\frac{n\cdot \sigma n}{N_{0}}, \end{array}$$

where *σ* is the Cauchy stress tensor and $N_{0}$ the pressure exerted by the ocean on the ice shelf:

$$\begin{array}{c} N_{0}=\frac{1}{2}\rho_{\mathrm{ice}}\left(1-\frac{\rho_{\mathrm{ice}}}{\rho_{\mathrm{water}}}\right)gH, \end{array}$$

where *H* is ice thickness, and the other constants in this equation are not relevant for our approach described below. Fürst (1) added to this definition of buttressing by defining Maximum Buttressing, where buttressing is maximized when n aligns with the direction of least principal stress. Following Fürst, we calculate the maximum buttressing number using the least principal Cauchy stress $\sigma_{2}$ and take $\sigma_{2}=2\tau_{2}+\tau_{1}$, where *τ* is the deviatoric stress tensor.

However, this definition of buttressing relies heavily on assumptions of ice rheology that are not well constrained (89, 90). The choice of ice softness parameter *A* when inferring stresses can dramatically impact the value of $K_{n}$, with certain choices of A eliminating negative values of $K_{n}$ entirely and other choices producing values of $K_{n}$ that are on the order of -10. With such a unique temporally resolute dataset, we are able to remove assumptions of ice rheology by defining a new Normalized Change in Buttressing Number, ${\tilde{K}}_{\Delta }$, relative to a reference state (subscript *ref*):

$$\begin{array}{c} {\tilde{K}}_{\Delta }=\frac{K_{n}-K_{n_{\mathrm{ref}}}}{1-K_{n_{\mathrm{ref}}}}. \end{array}$$

Simplifying this expression by canceling common terms, we arrive at:

$$\begin{array}{c} {\tilde{K}}_{\Delta }=1-\frac{H_{\mathrm{ref}}}{H}\frac{2{\dot{\epsilon }}_{2}+{\dot{\epsilon }}_{1}}{2{\dot{\epsilon }}_{2_{\mathrm{ref}}}+{\dot{\epsilon }}_{1_{\mathrm{ref}}}}{\left(\frac{{\dot{\epsilon }}_{E}}{{\dot{\epsilon }}_{E_{\mathrm{ref}}}}\right)}^{(1-n)/n}, \end{array}$$

which is unitless and independent of most assumed rheological parameters aside from the flow law exponent *n*. As shown in Fig. 6, the choice of *n* does not substantially impact the value of ${\tilde{K}}_{\Delta }$. However, the choice of reference state does impact the values produced. In the main text, we chose 2015 as the reference state and provide *SI Appendix*, Figs. S6 and S7 that show how the values of ${\tilde{K}}_{\Delta }$ vary with choice of reference state. One challenge with this methodology is that very small strain rates can lead to ${\tilde{K}}_{\Delta }$ blowing up. To mitigate this issue, we filtered the calculated buttressing number using the MATLAB function medfilt2, which sets the value of a pixel to the median of the 3 × 3 square of pixels surrounding it.

## Supplementary Material

Appendix 01 (PDF)

Movie S1. Change in ice velocity relative to 2015 over Pine Island Ice Shelf from 2015 to 2024. Grey represents regions where ice velocity decreases relative to 2015 values. The velocity increase initiates from the SSM after the 2017 calving event and propagates towards the NSM. After the 2018 and 2020 calving events, the velocity increase propagates from the NSM towards the SSM. The plot below the map shows velocities averaged over the black box in the center of the ice shelf, with vertical black bars denoting calving events. Calving fronts are traced by the authors from Sentinel-1 imagery. Grounding line from (1, 2).

Movie S2. Evolution of ${\dot{\epsilon }}_{E}$ on Pine Island Ice Shelf from 2015 to 2024. The plot below the map shows ${\dot{\epsilon }}_{E}$ averaged over the black box in the center of the ice shelf, with vertical black bars denoting calving events. Calving fronts are traced by the authors from Sentinel-1 imagery. Grounding line from (1, 2).

Movie S3. Change in ${\dot{\epsilon }}_{E}$ relative to 2015 over Pine Island Ice Shelf from 2015 to 2024. Green denotes areas where ${\dot{\epsilon }}_{E}$ increases relative to 2015 and blue denotes areas where ${\dot{\epsilon }}_{E}$ decreases relative to 2015. As the margins weaken, a distinctive pattern develops of decreasing ${\dot{\epsilon }}_{E}$ just outside of the margin and increasing ${\dot{\epsilon }}_{E}$ within the margin. The plot below the map shows ${\dot{\epsilon }}_{E}$ averaged over the black box in the center of the ice shelf, with vertical black bars denoting calving events. Calving fronts are traced by the authors from Sentinel-1 imagery. Grounding line from (1, 2).

Movie S4. Evolution of ${\dot{\epsilon }}_{2}$ on Pine Island Ice Shelf from 2015 to 2024. Red denotes areas of positive ${\dot{\epsilon }}_{2}$ where the ice shelf is in pure tension. After the 2020 calving event, the majority of the ice shelf transitions to a purely tensile regime. The plot below the map shows ${\dot{\epsilon }}_{2}$ averaged over the white box in the center of the ice shelf, with vertical black bars denoting calving events. Calving fronts are traced by the authors from Sentinel-1 imagery. Grounding line from (1, 2).

## Data Availability

Derived velocity and strain rate data are available on PANGAEA at https://doi.org/10.1594/PANGAEA.992895 (91). Calving front traces are available on Zenodo at https://doi.org/10.5281/zenodo.18961392 (44). Grounding lines used in all figures are from the RINGS/Bedmap3 grounding line of the Antarctic Ice Sheet dataset, available through the following citations: (40, 41, 92). Sentinel-1 GRD data were obtained and processed via the Alaska Satellite Facility’s Vertex tool. Fig. 6 contains DINSAR-derived grounding line data from MEaSUREs, available via the following citations: (24, 93). Thickness data used for buttressing calculations are from refs. 50 and 68. All software used in this analysis are available through their referenced sources.
