# Supplementary material for: Near-total loss of buttressing stresses observed on Pine Island Ice Shelf, West Antarctica
Source: Proc Natl Acad Sci U S A. 2026 Aug 11;123(33):e2602994123. doi: 10.1073/pnas.2602994123 (PMC13486552; doi:10.1073/pnas.2602994123)
Supplement: Supplementary file 1 [file pnas.2602994123.sapp.pdf]

Supporting Information for

Near-total loss of buttressing stresses observed on Pine Island Ice Shelf, West Antarctica

Sarah Wells-Moran, Brent Minchew, and Bryan Riel

Sarah Wells-Moran.
E-mail: swellsmo@uchicago.edu

This PDF file includes:

Figs. S1 to S9
Legends for Movies S1 to S4
SI References

Other supporting materials for this manuscript include the following:

Movies S1 to S4

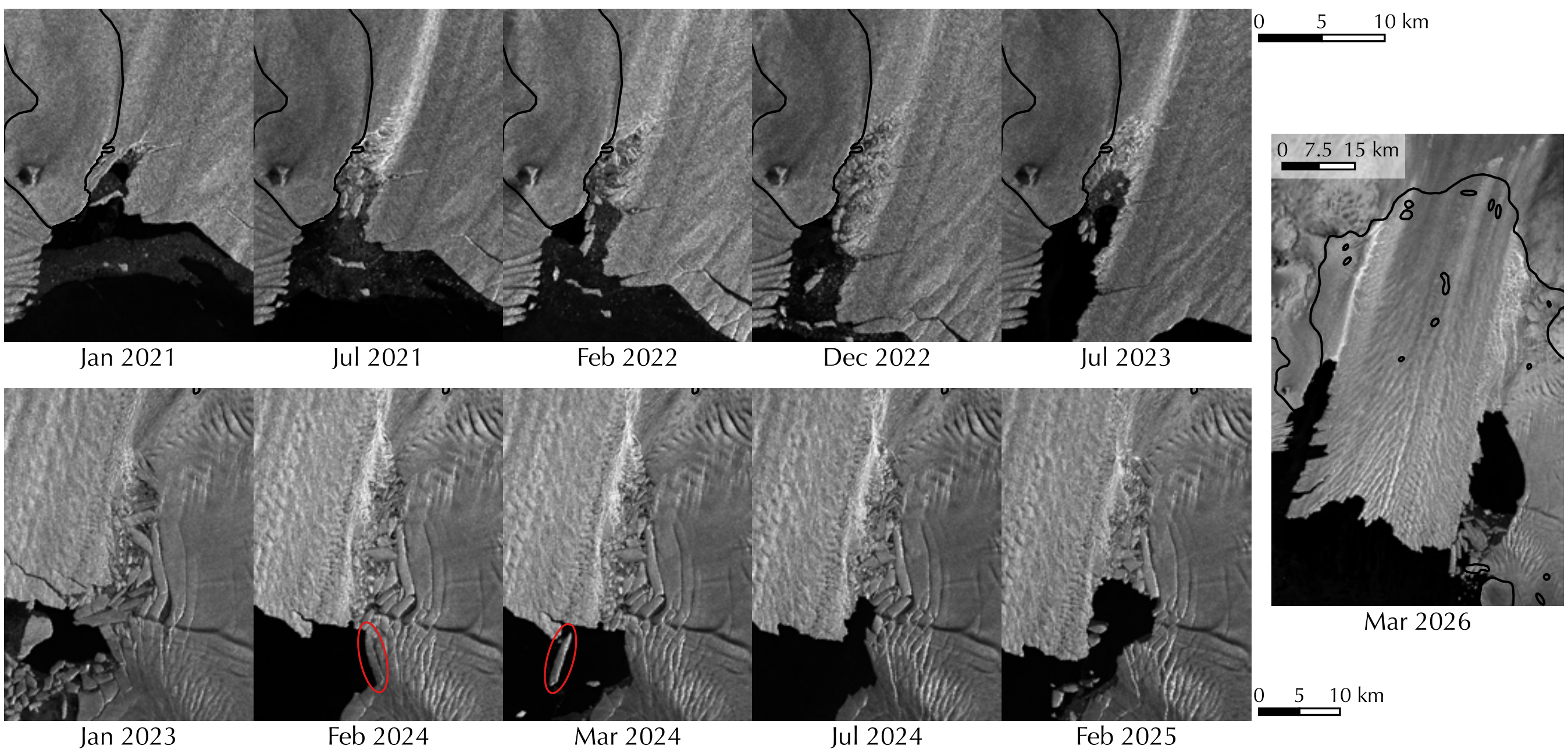

Fig. S1. (Top) Melange steadily generated and evacuated from the NSM. Melange generation stops in late 2023, leaving open water after that date. **(Bottom)** Melange stuck in the SSM until a small iceberg breaks off of the PIG Southern Ice Shelf (circled in red), after which melange begins evacuating. **(Right)** Open water present along 50% of the central trunk margins of PIIS in March 2026

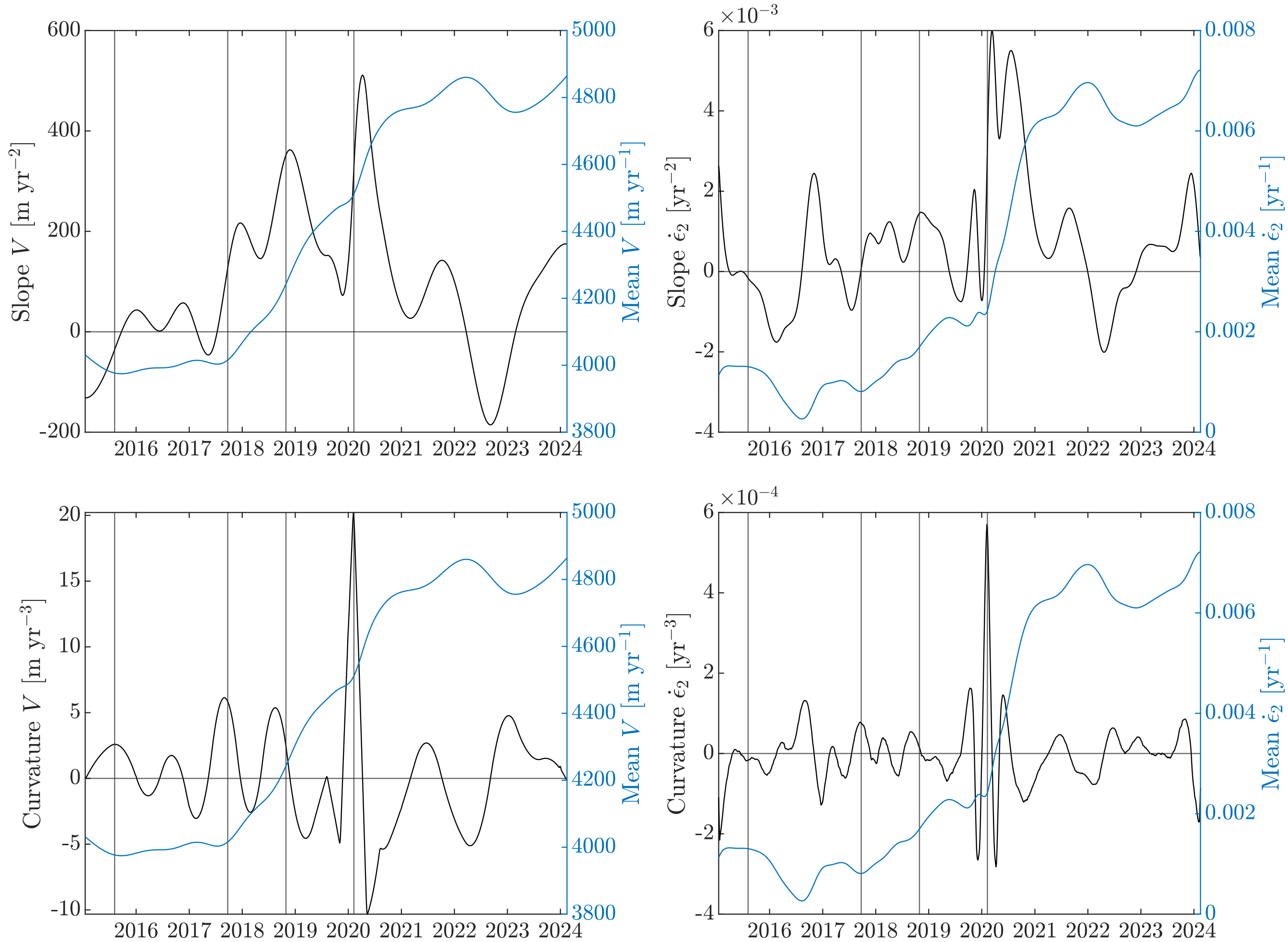

Fig. S2. The **(top row)** slope and **(bottom row)** curvature of mean velocity magnitude (V) and mean least principal strain rate ($\dot{\epsilon}_2$) versus time. The greatest slope and curvature in both least principal strain rate and ice speed occurs around the 2020 calving event. Speeds increase from 2017 through 2022. Least principal strain rates increase slowly after $\mathbf{C}_{2017}$ and then almost triple between 2020 and 2022. After C_{2020}, the second largest peak in flow speed curvature occurs just prior to $\mathbf{C}_{2017}$ and another similarly-sized peak occurs preceding $\mathbf{C}_{2018}$, although these peaks are of similar magnitude to peaks that occur in 2021 and 2023 when no calving events occur.

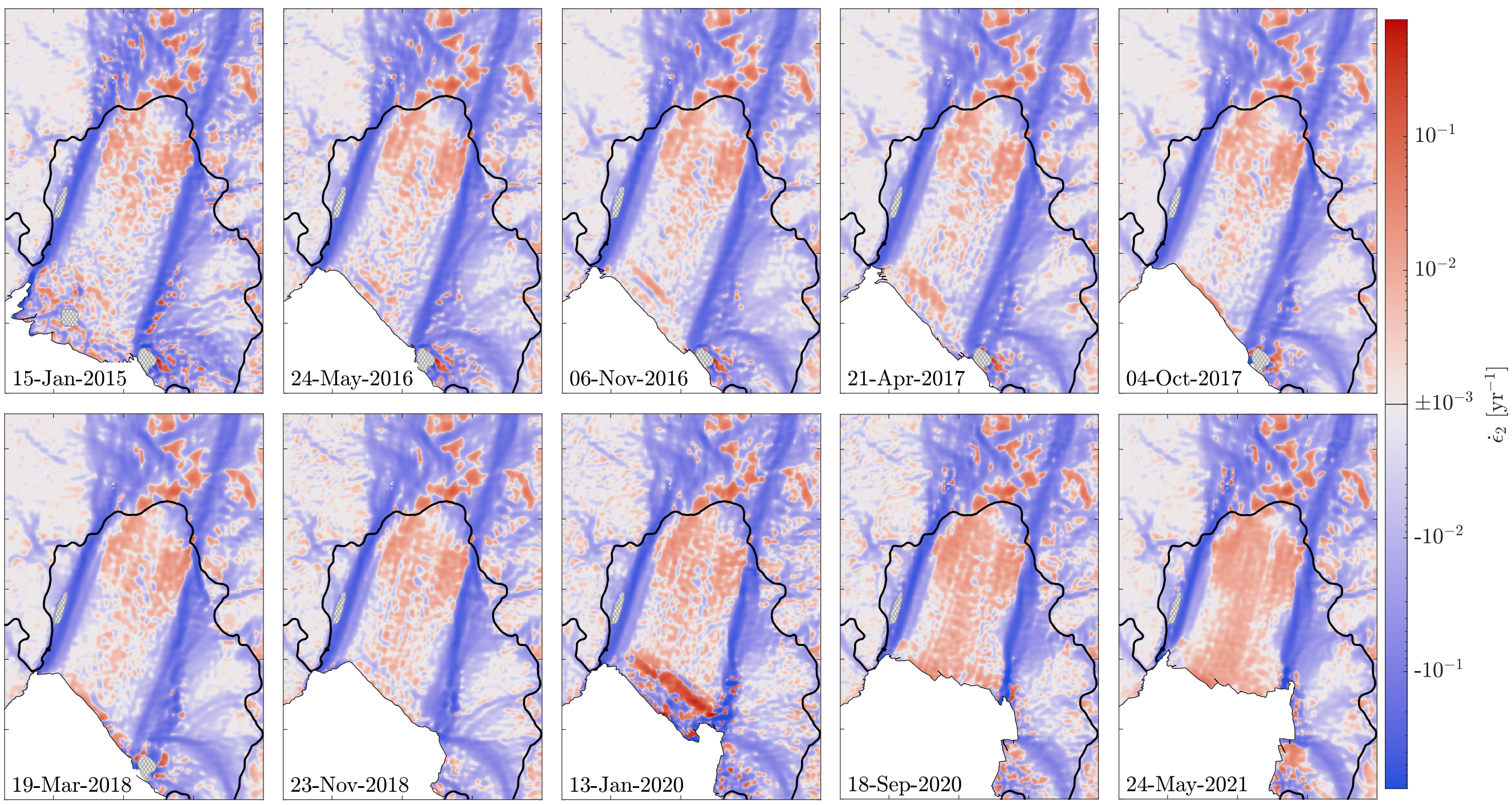

Fig. S3. (Top) $\dot{\epsilon}_2$ fields prior to loss of buttressing **(Bottom)** Slight increase in area of tensile regime ($\dot{\epsilon}_2 > 0$) after $\mathbf{C}_{2017}$, followed by a transition to a near-total tensile regime across the entire ice shelf after $\mathbf{C}_{2020}$

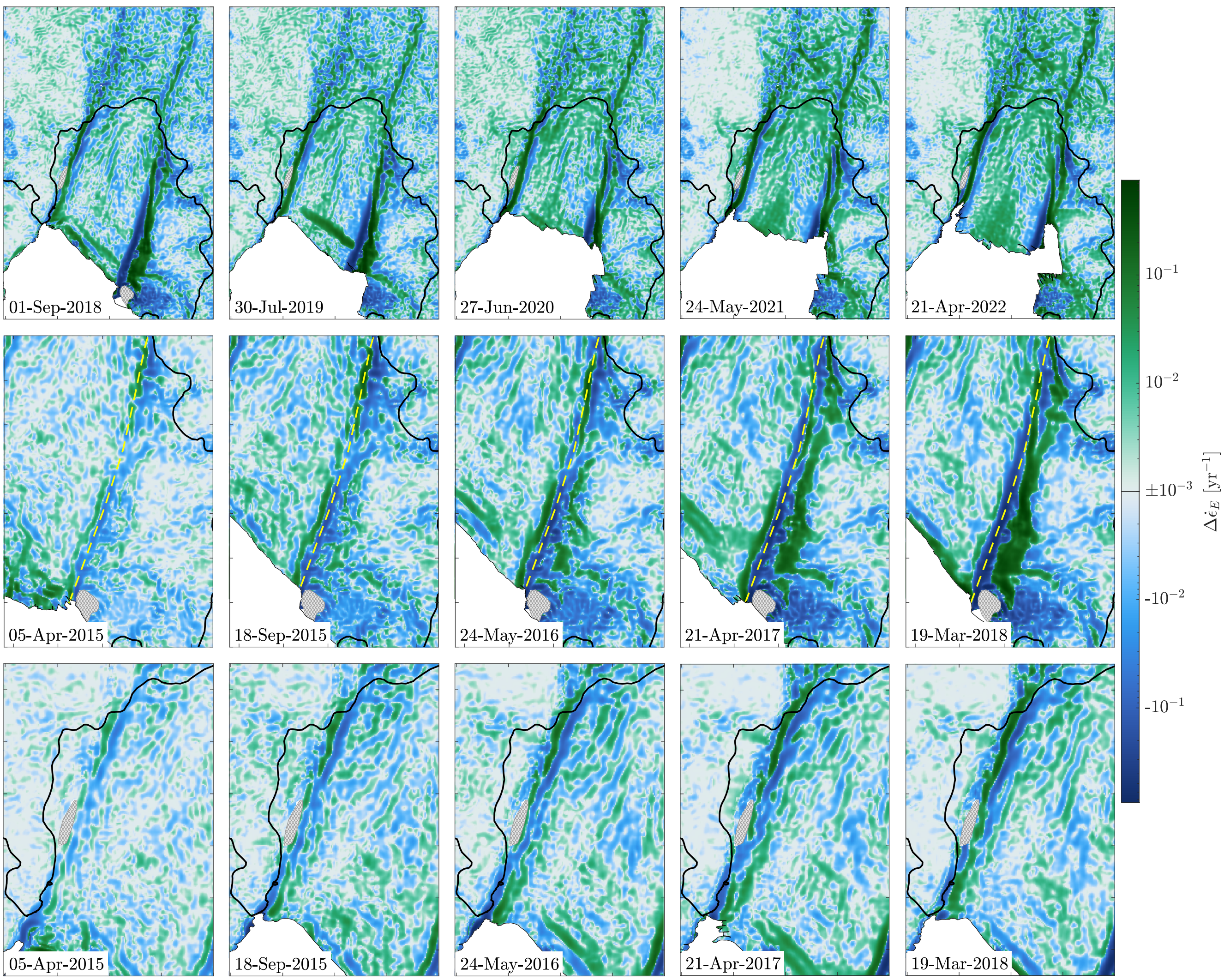

Fig. S4. Change in effective strain rates relative to January 2015 in the **(Bottom)** Northern Shear Margin and **(Middle)** Southern Shear Margin between 2015 and 2018, and **(Top)** the continued evolution and intensification of strain rates across the entire ice shelf from 2018 onwards. Between 2015 and 2018, the SSM migrates 5-10 km outwards from its initial location (yellow dashed line, traced from satellite imagery), accompanied by increasing damage and crevassing in the margin. As the margin weakens and migrates, it leaves a distinctive pattern in the $\Delta\dot{\epsilon}_E$ fields of bands of decreasing strain rates adjacent to bands of increasing strain rates. This pattern can be used to locate other regions where margins are weakening. This same pattern can be seen developing towards the grounding line of the NSM and lengthening downstream as the SSM migrates.

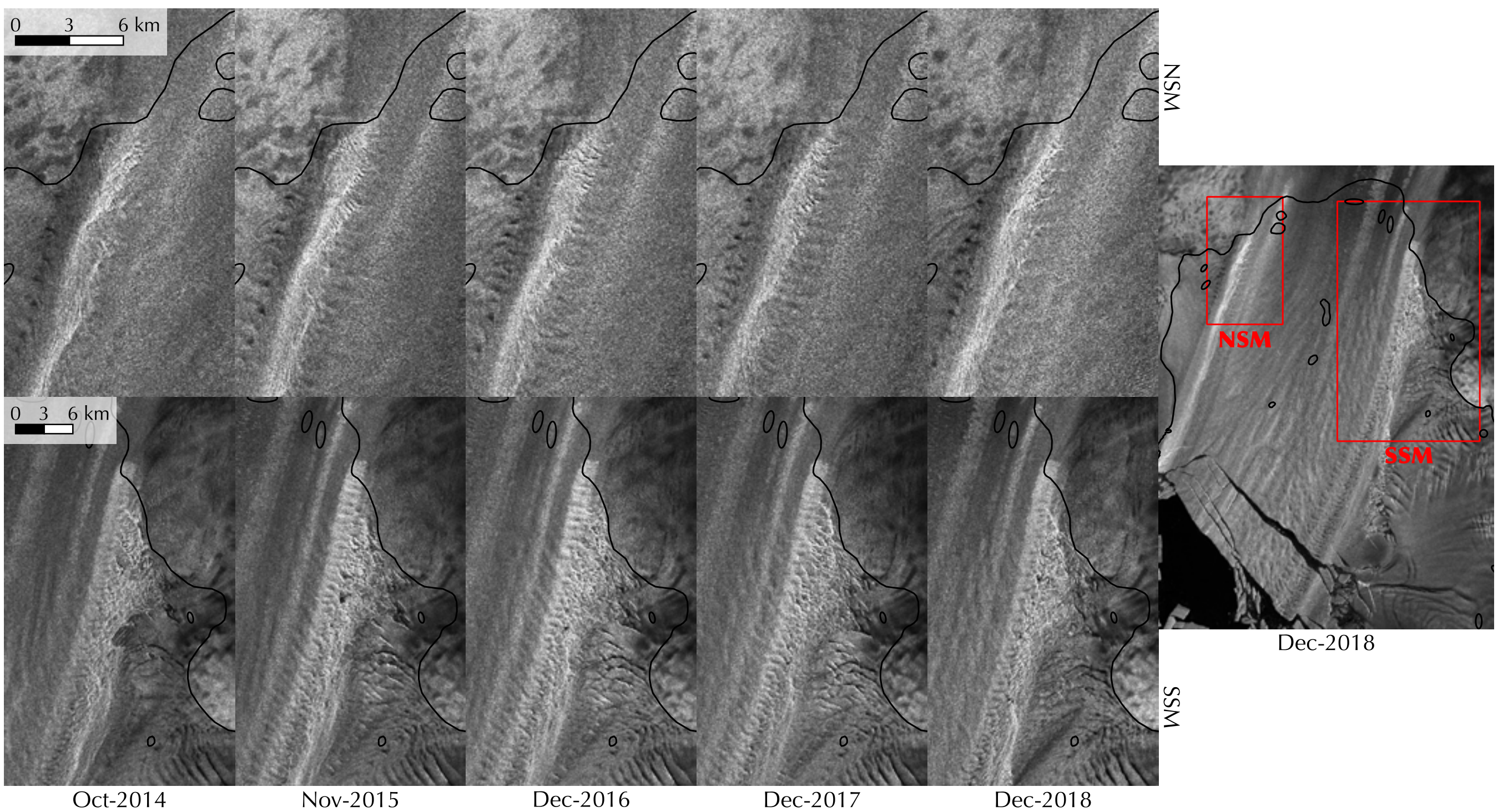

Fig. S5. Regions which generate damage near the grounding line in both the **(Top)** NSM and **(Bottom)** SSM. The damage then advects downstream with ice flow. Damage generation within the SSM is likely to have been occurring for 5-10 years prior to 2015, as evidenced by the large area of damage downstream of the grounding line. The damage along the NSM is likely to have initiated relatively recent to 2015. Contains modified Copernicus Sentinel-1 imagery

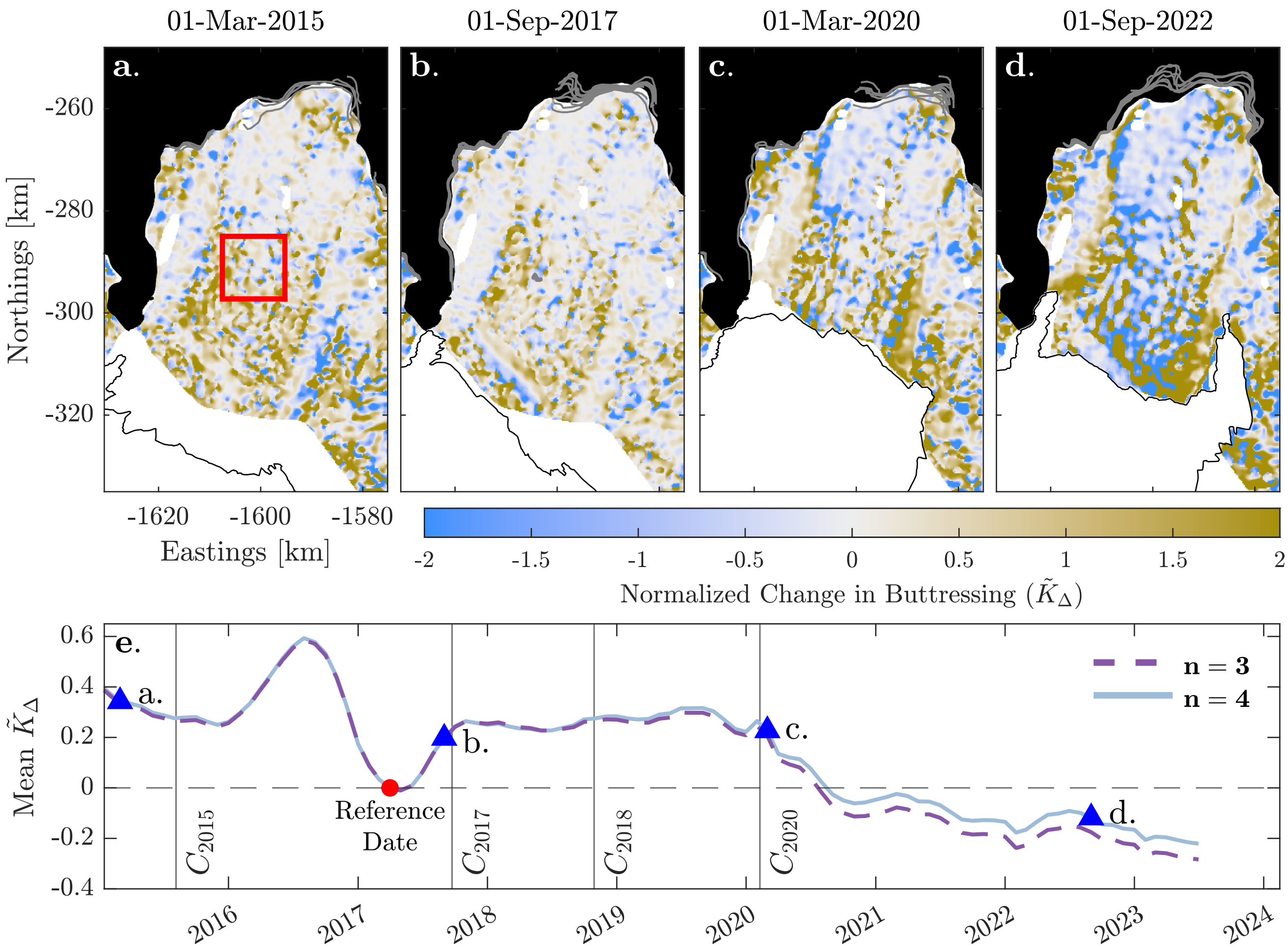

Fig. S6. Similar to Figure 6 in the main text except we calculate normalized change in buttressing ($\tilde{K}_\Delta$) relative to 2017

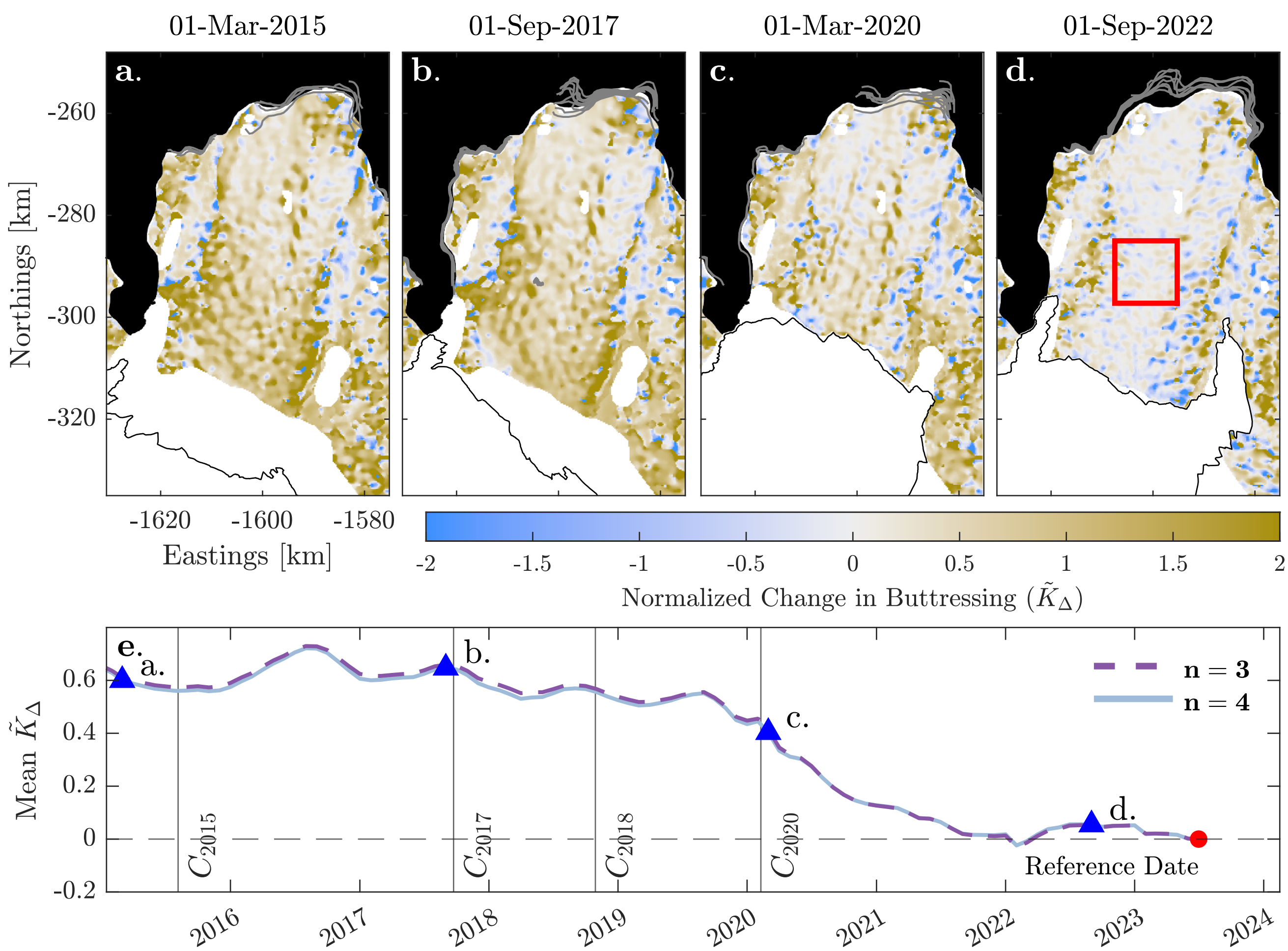

Fig. S7. Similar to Figure 6 in the main text except we calculate normalized change in buttressing ($\tilde{K}_\Delta$) relative to 2023

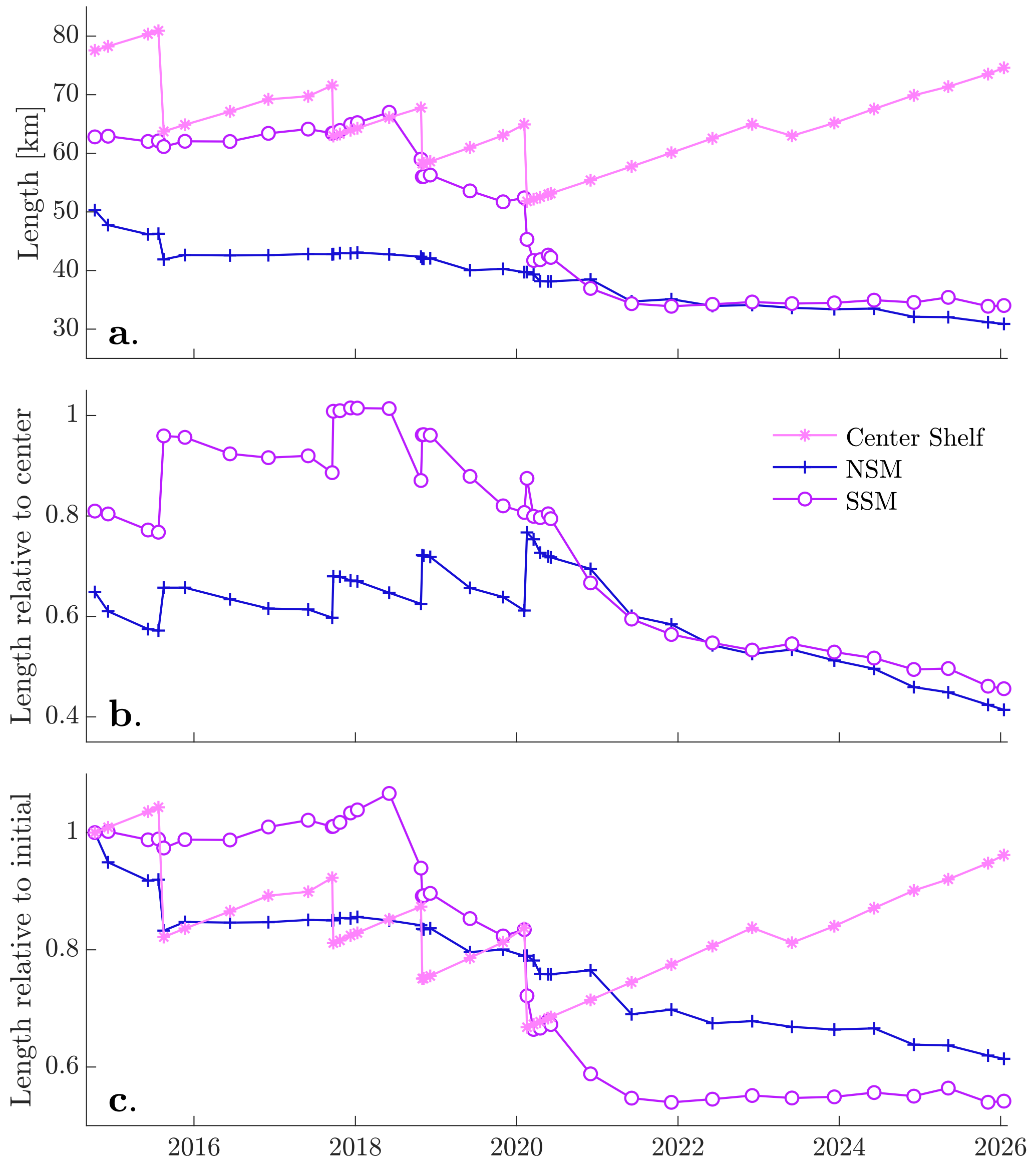

Fig. S8. **(Top)** Total length from the grounding line of the furthest extents of the center of the ice shelf, the NSM, and the SSM. **(Middle)** Length of the NSM and SSM relative to the length of the center shelf. **(Bottom)** Length of the NSM, SSM, and central shelf relative to the initial measured lengths.

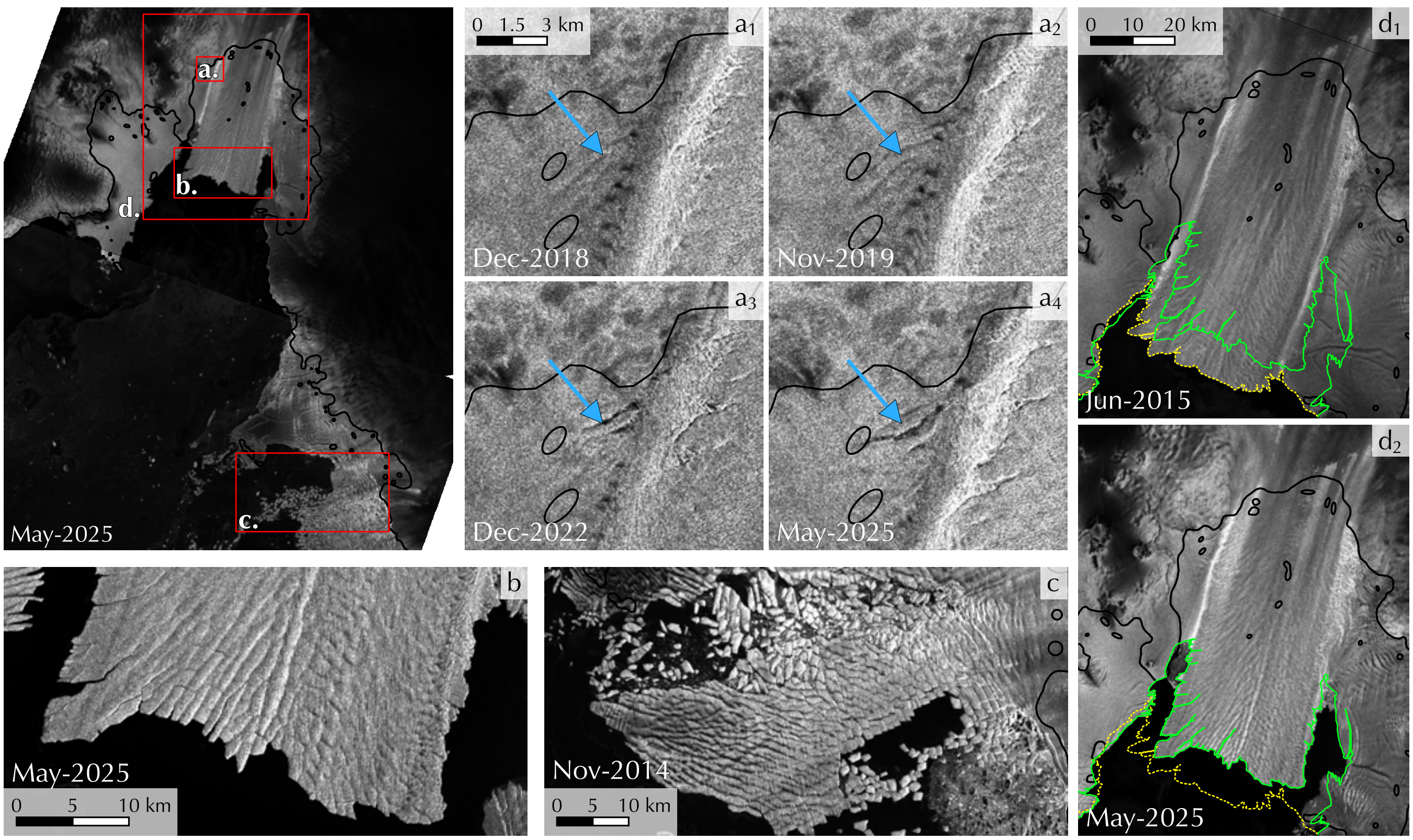

Fig. S9. **(a)** The evolution of a new rift (identified with a blue arrow) close to the grounding line in the NSM, which could contribute to the next calving event on PIIS. **(b)** A distinctive scale-like pattern has developed on the PIIS after the disintegration of both shear margins, which resembles the pattern observed on the **(c)** Thwaites Western Ice Tongue prior to breakup. **(d)** The 2015 calving front (dashed yellow line) and the 2025 calving front (solid green line) overlain on (d_1) 2015 and (d_2) 2025 Sentinel imagery, showing the rotation of the PIIS trunk after the loss of contact with the Southern ice shelf. Contains modified Copernicus Sentinel-1 imagery

Movie S1. Change in ice velocity relative to 2015 over Pine Island Ice Shelf from 2015 to 2024. Grey represents regions where ice velocity decreases relative to 2015 values. The velocity increase initiates from the SSM after the 2017 calving event and propagates towards the NSM. After the 2018 and 2020 calving events, the velocity increase propagates from the NSM towards the SSM. The plot below the map shows velocities averaged over the black box in the center of the ice shelf, with vertical black bars denoting calving events. Calving fronts are traced by the authors from Sentinel-1 imagery. Grounding line from (1, 2)

Movie S2. Evolution of $\dot{\epsilon}_E$ on Pine Island Ice Shelf from 2015 to 2024. The plot below the map shows $\dot{\epsilon}_E$ averaged over the black box in the center of the ice shelf, with vertical black bars denoting calving events. Calving fronts are traced by the authors from Sentinel-1 imagery. Grounding line from (1, 2)

Movie S3. Change in $\dot{\epsilon}_E$ relative to 2015 over Pine Island Ice Shelf from 2015 to 2024. Green denotes areas where $\dot{\epsilon}_E$ increases relative to 2015 and blue denotes areas where $\dot{\epsilon}_E$ decreases relative to 2015. As the margins weaken, a distinctive pattern develops of decreasing $\dot{\epsilon}_E$ just outside of the margin and increasing $\dot{\epsilon}_E$ within the margin. The plot below the map shows $\dot{\epsilon}_E$ averaged over the black box in the center of the ice shelf, with vertical black bars denoting calving events. Calving fronts are traced by the authors from Sentinel-1 imagery. Grounding line from (1, 2)

Movie S4. Evolution of $\dot{\epsilon}_2$ on Pine Island Ice Shelf from 2015 to 2024. Red denotes areas of positive $\dot{\epsilon}_2$ where the ice shelf is in pure tension. After the 2020 calving event, the majority of the ice shelf transitions to a purely tensile regime. The plot below the map shows $\dot{\epsilon}_2$ averaged over the white box in the center of the ice shelf, with vertical black bars denoting calving events. Calving fronts are traced by the authors from Sentinel-1 imagery. Grounding line from (1, 2)

References

1. G Moholdt, H Pritchard, J Maton, British Antarctic Survey, RINGS/Bedmap3 grounding line of the Antarctic Ice Sheet (2025).
2. K Matsuoka, et al., Towards an improved understanding of the Antarctic coastal zone and its contribution to future global sea level (2025).
